# Imprinting bulk amorphous alloy at room temperature

**DOI:** 10.1038/srep16540

**Published:** 2015-11-13

**Authors:** Song-Yi Kim, Eun-Soo Park, Ryan T. Ott, Thomas A. Lograsso, Moo-Young Huh, Do-Hyang Kim, Jürgen Eckert, Min-Ha Lee

**Affiliations:** 1 Rare Metals R&D Group, Korea Institute of Industrial Technology, Incheon 406-840, Korea; 2 Global Technology Center, Samsung Electronics Co., Ltd., Suwon, 443-742, Korea; 3 Division of Materials Sciences and Engineering, Ames Laboratory (USDOE), Ames, Iowa 50011, USA; 4 Department of Materials Science and Engineering, Korea University, Seoul, 136-701, Korea; 5 Department of Metallurgical Engineering, Yonsei University, Seoul, 120-749, Korea; 6 Erich Schmid Institute of Materials Science, Austrian Academy of Sciences, Jahnstraße 12, A-8700 Leoben, Austria; 7 Department Materials Physics, Montanuniversität Leoben, Jahnstraße 12, A-8700 Leoben, Austria

## Abstract

We present investigations on the plastic deformation behavior of a brittle bulk amorphous alloy by simple uniaxial compressive loading at room temperature. A patterning is possible by cold-plastic forming of the typically brittle Hf-based bulk amorphous alloy through controlling homogenous flow without the need for thermal energy or shaping at elevated temperatures. The experimental evidence suggests that there is an inconsistency between macroscopic plasticity and deformability of an amorphous alloy. Moreover, imprinting of specific geometrical features on Cu foil and Zr-based metallic glass is represented by using the patterned bulk amorphous alloy as a die. These results demonstrate the ability of amorphous alloys or metallic glasses to precisely replicate patterning features onto both conventional metals and the other amorphous alloys. Our work presents an avenue for avoiding the embrittlement of amorphous alloys associated with thermoplastic forming and yields new insight the forming application of bulk amorphous alloys at room temperature without using heat treatment.

Thermo-mechanical forming and shaping treatments are well known metallurgical processes that are employed on conventional crystalline metallic alloys like steels, brass, etc. in order to exploit their use for engineering applications^1^,^2^,^3^. However, crystalline metallic materials are not ideally suited for high precision parts due to the limitation of surface roughness by their grains. Amorphous alloys, especially metallic glasses (MGs), are highly attractive for fine-scale imprinting or patterning materials due to their exceptional mechanical properties combined with the fact that they are intrinsically free from grain boundaries^3^,^4^,^5^,^6^,^7^. Thermo-plastic forming (TPF) processes of metallic glasses in the supercooled liquid region (between glass transition temperature, T_g_, and crystallization temperature, T_x_) via homogeneous flow has been the most commonly exploited method for forming glassy metals. For instance, Saotome *et al*. developed a method for the configuration of three-dimensional structures by micro-forming of Pt-based metallic glass^8^,^9^. Kumar *et al*. obtained atomistic surface smoothness through TPF of Pt-based metallic glass, Hasan *et al*. demonstrated patterning of Pt-based metallic glass by TPF for functional application and Chu *et al*. showed nanoimprinting of gratings on Pd-based metallic glass by TPF process, respectively^10^,^11^. Moreover, achievable strain rates and significant shapes through thermoplastic blow molding of Zr-based metallic glasses and Pt-based metallic glass for the fabrication of hemispherical shell resonators were reported by Schroers *et al*.^12^,^13^. Additionally, Chiu *et al*. explored effect of processing parameters on thermoplastic extrusion method to create objects of Zr-based metallic glass^14^.

The plastic flow of bulk amorphous alloy at room temperature (RT), however, typically occurs inhomogeneously and is localized into narrow shear bands resulting in catastrophic failure^4^,^5^,^6^,^7^,^15^. As a result, bulk amorphous alloys show macroscopic brittleness or limited plasticity compared to crystalline alloys at room temperature. In order to increase the reliability of bulk amorphous alloys, enhancement of plasticity is a crucial issue for their utilization in engineering applications. It is widely accepted that the most common ways to achieve substantial deformability of bulk amorphous alloy as required in shaping applications is to: 1) Add a ductile second phase (e.g., crystalline particles)^16^,^17^, 2) confine the deformation geometry so that shear bands cannot propagate through the sample and cause failure^3^,^18^ or 3) increase the processing temperature into or near the supercooled liquid region, thus utilizing viscous flow (homogenous deformation) behavior at elevated temperatures, i. e., TPF^2^,^3^. The major limitations with approaches 1 and 2 are they rely on the profuse generation of shear bands, which is detrimental to the surface in forming applications^2^. Approach 3 can prevent the formation of shear bands, however, when an amorphous alloy is reheated above or near its glass transition temperature, T_g_, it will relax into the metastable supercooled liquid and eventually crystallize. In particular, the embrittlement caused both by sub-T_g_ annealing (structural relaxation) and by above-T_g_ annealing. The embrittlement caused by sub-T_g_ annealing can be restored or avoided during TPF by a short anneal above-T_g_, however, these thermal processes can be difficult to control^19^. Instead, crystallization by excess annealing has been identified as the source of above-T_g_ embrittlement, and even the early stages of the crystallization process often leads to significant embrittlement^19^,^20^,^21^.

It is important to distinguish between enhanced plasticity and homogenous flow when discussing the room temperature deformation of glassy metal alloys. As discussed above, enhanced plasticity can be achieved by distributing the strain over numerous shear bands, however, the flow is still inhomogeneous since it is localized in shear bands. For example, recent works show amorphous metal alloys tested at slow strain rates can achieve large macroscopic plastic strains at RT if the sample aspect ratio (height/diameter) is kept low (0.9), however, their deformation behavior is still basically governed by shear localization with multiple shear bands^18^,^22^. In contrast, homogeneous deformation does not appear to show any localization of the strain beyond the atom-scale. It is exploited by Spaepen that deformation of bulk metallic glass (BMG) could be homogeneous deformation at room temperature under very slow deformation^4^. More recent work has observed homogenous deformation at room temperature under high, close to the yield strength, stresses^22^,^23^,^24^,^25^. Since homogeneous deformation in amorphous alloys is dependent on the both the strain rate and the temperature, we must consider the ratio of test temperature to glass transition temperature (reduced temperature) for a given alloy^4^,^23^. Recently, Ce- and Zn-based amorphous alloys have been reported to exhibit homogeneous deformation at RT without shear localization. Interestingly, these systems have very low glass transition temperature (<75 K above room temperature), which may account for the transition to homogeneous flow at slow strain rates^24^,^25^. It should also be noted that reducing the size of a monolithic amorphous metal below about 100 nm can also lead to homogeneous flow, but here we are not considering size effects^26^.

For monolithic metallic glasses, it is well-known that certain alloys such as Fe-based and Hf-based alloys have inherently brittle behavior with essentially zero macroscopic plasticity, while others (i.e., Zr-based and Pt-based alloys) exhibit large plastic strains in compression, bending, or under intensely confining pressure^27^,^28^. The brittle nature of amorphous alloys has limited their use in engineering application, however, we have found that there is an inconsistency between macroscopic plasticity and deformability of brittle amorphous alloy. Specifically, these brittle amorphous alloys can be deformed at room temperature without shear localization or fracture. In the current study, we report on patterning by cold-plastic deformation of a typical brittle bulk glassy alloy by homogeneous plastic flow without shear fracture at RT.

## Results

**Figure 1:**
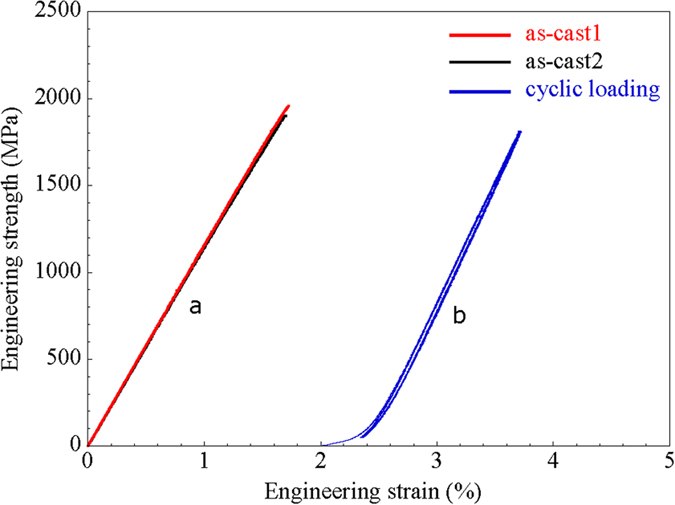
(**a**) Engineering stress strain curve obtained from the uniaxial compression test for the as-cast Hf-based amorphous alloy with initial strain rate: 3 × 10^−4^ s^−1^. (**b**) Engineering stress strain curve obtained from the constraint 30 times cyclic compression test for the as-cast Hf-based amorphous alloy machine stop at 90% of maximum stress without fracture and initial strain rate is 3 × 10^−4^ s^−1^.

A Hf-based amorphous alloy with nominal composition Hf_44.5_Cu_27_Ni_13.5_Ti_5_Al_10_ (at%) was selected for the current study because this alloy has high glass-forming ability and generally exhibits brittle (zero macroscopic plasticity) deformation characteristics^17^. We performed both uniaxial compression tests and cyclic compressive deformation at a constant strain rate of 3 × 10^−4^ s^−1^ at RT using an Instron testing machine in order to verify the brittleness of the monolithic glassy phase. The uniaxial compression test results shown in Fig. 1(a) clearly reveal that the as-cast amorphous alloy exhibits a typical brittle behavior and fails without any macroscopic plastic strain. To examine possible changes of the mechanical properties or structure associated with repeated elastic straining, we performed cyclic compression tests below the yield stress of the alloy (93% of the yield strength; 1813 MPa) under load-controlled conditions at RT, Fig. 1(b). As with the constant strain rate experiments, the compressive plasticity of the specimens was estimated to be zero, confirming that the monolithic Hf-based amorphous alloy exhibits intrinsic brittleness^17^.

Interestingly, the monolithic Hf-based amorphous alloy exhibits a hysteresis during the cyclic loading but the strain accumulation is almost negligible from the width of the hysteresis loop. There is no difference between the common points and the unloading strain values up to 30 cycles as shown in Fig. 1(b). Hysteresis is a typical characteristic feature of viscoelastic behavior rather than purely elastic behavior. It is well known that viscoelasticity depends on the degree of structural reconfiguration and from the movement of atoms or clusters in the glassy phase^29^,^30^,^31^,^32^,^33^. The existence of the interval of the hysteresis loop, as shown in Fig. 1(b), means that the atoms or clusters will move into more stable sites with energy proportional to the area of the loop by the structural rearrangement during the individual loading cycles^30^,^31^,^32^.

**Figure 2:**
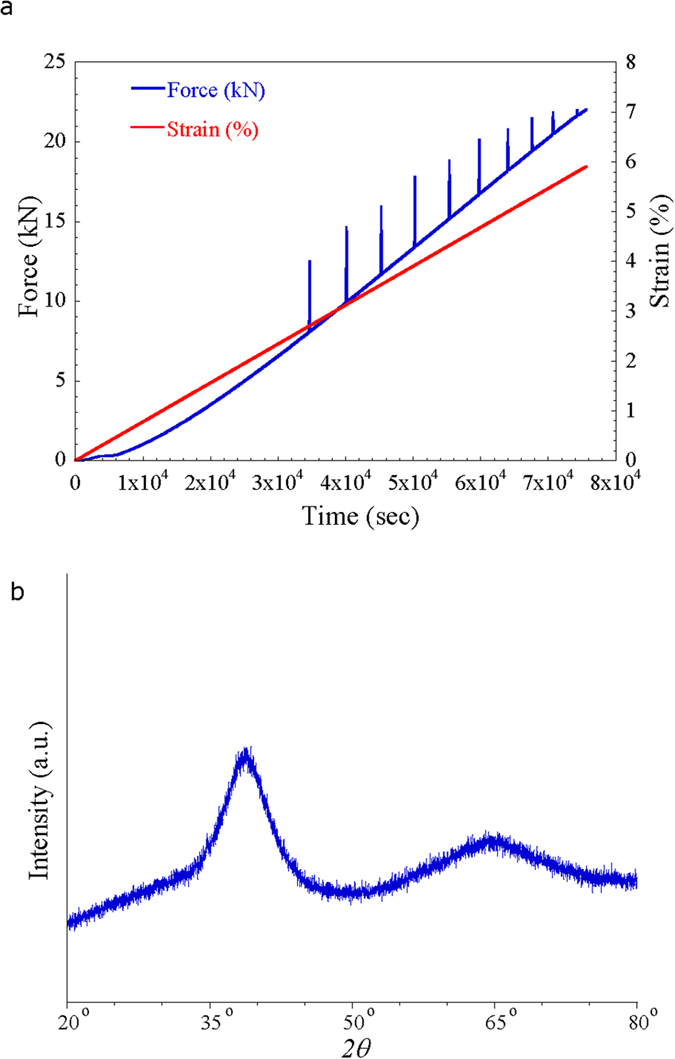
(**a**) Load (kN) as a function of displacement rate (1/s) for the uniaxial compressive loading for patterning and strain (%) as a function of time for the uniaxial compressive loading for patterning. (**b**) X-ray diffraction pattern obtained from cold-patterned Hf-based amorphous alloy with long time scan by (2θ = 0.08°)/step speed.

Figure 2(a) is plot of load/strain versus loading time from which the load and total displacement during the test. The spikes in load clearly appeared later in the test, suggesting accumulation of local strain to initiate atomic movements. However, the plot of the strain versus time shows that strain continuously increases without any fluctuation. Figure 2(a) shows the stress-strain curve obtained from the Hf-based amorphous alloy by loading continuously up to 75% of maximum strength (1453 MPa) at a low strain rate of 1.5 × 10^−5^ s^−1^. The plot represents a typical uniaxial compressive test result of brittle amorphous alloy without any plasticity, however, examination of the Hf-based amorphous sample after unloading reveals that is plastically compressed to 5% based on measurements of the gage length (h_as−cast_ = 1.55 mm, h_deformed_ = 1.47 mm, ∆h_deformation_ = 80 μm) without any obvious signs of cracking or shear banding. The total displacement is larger than actual sample deformation amounts due to the compliances of test equipment. The large increases in stress seen in Fig. 2(a) were observed in multiple samples. To verify the jumps in stress were not due to fluctuations in the load frame, the stress as a function of time was investigated for one of the spikes as seen in Fig. 2(a). Interestingly, the stress shows a rapid increase followed by duration of 2.7 min with pop-up stress difference levels 93 ~ 311 MPa. This time dependence of the stress relaxation strongly suggests that the spikes are due to deformation induced structural rearrangements. Similar sharp stress increases or serrate flow above the yield stress of metallic glass under compressive loading are observed in Zr-based and Pd-based MG systems^34^,^35^. It might be that serrated flow correlated with atomic rearrangement or shear localization, however, despite extensive studies, the physical origin of serrated flow as well as its connection to the shear banding in MG is still unclear.

As discussed above, glassy metal alloys with low glass transition temperatures (e.g., Ce- and Zn-based) have been reported to exhibit homogeneous flow at room temperature^24^. The homogeneous nature of the amorphous alloy in these low T_g_ alloys can be better understood by looking at the reduced temperature (T/T_g_) that deformation is imparted to specimen. For room temperature (T = 298 K), T/T_g_ is above 0.8 for the Ce- (0.82) and Zn-based (0.93) alloys. In contrast, the T_g_ of the Hf-based amorphous alloy is 787 K, and thus, T/T_g_ for room temperature deformation corresponds to 0.37 which has similar value to Fe-based amorphous alloys (0.38), well within the regime in which plastic deformation is expected to be inhomogeneous^4^,^18^,^24^. Ke *et al*. have reported that a Zr-based alloy deformed elasto-statically at room temperature (similar T/T_g_ to the Hf-based alloy) also exhibits homogeneous flow^22^. The major component of the inelastic strain, however, was anelastic strain, which was recovered after removing the load. In contrast, we have found that the inelastic strain in the Hf-based alloy is predominantly plastic strain since it is not recovered after unloading.

**Figure 3:**
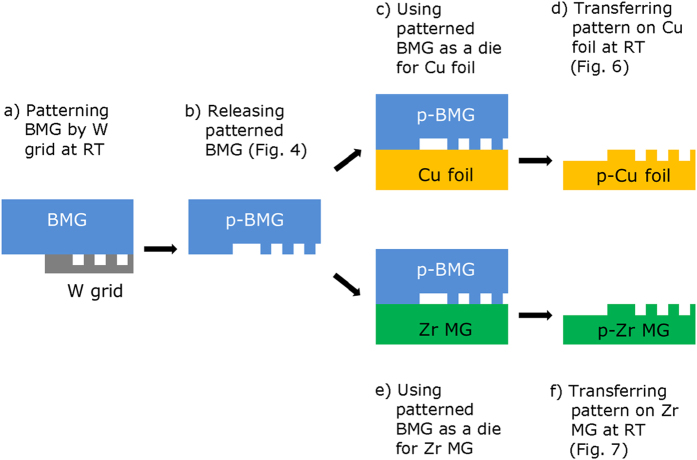
The schematic illustration on the concept of a processing for the patterning of Hf-based bulk metallic glass (BMG) and transferring of pattern on Cu metal and Zr-based metallic glass (MG) at room temperature.

The X-ray diffraction (XRD) pattern of the cold-patterned Hf-based amorphous alloy in Fig. 2(b) shows the typical broad maxima characteristic for amorphous materials and no distinct crystalline peaks are detected within the sensitivity limits of XRD. This result corroborates that the amorphous nature of the as-cast bulk glassy alloy does not devitrify during deformation or cold-patterning processes. From the uniaxial compression performed at room temperature, we see that macroscopic plastic deformation can be imparted without any evidence of slip steps at the surface, which are characteristic of shear bands. This absence of typical inhomogeneous deformation mechanisms makes room temperature patterning of the alloys a viable process. To date, micro-patterning of brittle bulk amorphous such as the Hf-based alloy is known only to be possible for temperatures near T_g_. To explore the possibility of room temperature patterning via cold plastic forming, rectangular shaped Hf-based amorphous alloy specimens, 4 mm in width and 2 mm height (aspect ratio = 0.5), were prepared from as-cast ingot. The ends of the samples were carefully polished flat and normal to the longitudinal axis to assure uniform loading in compression for patterning. As shown in Fig. 3, a tungsten die with a grid pattern was placed between the WC platen and the samples which were loaded at a strain rate of 1.5 × 10^−5^ s^−1^ up to 75% of the yield strength of amorphous alloy (1453 MPa). Since this strain rate is considerably slower than the quasi-static strain rate (3 × 10^−4^ s^−1^) utilized in the tests shown in Fig. 1(a), achieving the maximum stress requires 21 hours. Upon reaching the stress corresponding to 75% of the yield strength of the Hf-based amorphous alloy, the load was immediately removed.

**Figure 4:**
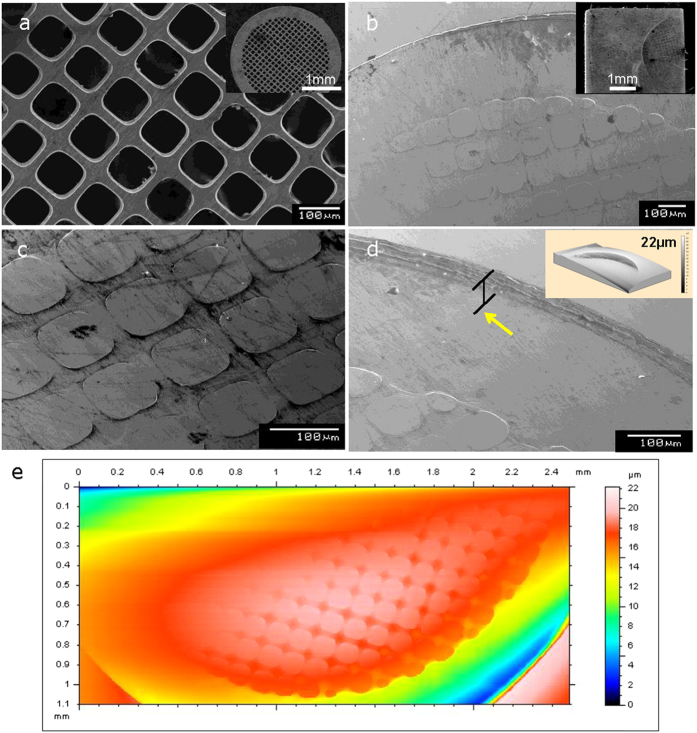
(**a**) SEM secondary electron image of tungsten net-pattern grid die with 97 μm pit spacing, 25 μm thickness produced by electro-deposition. (Pacific Grid-Tech); inset image shows macro overview of the SEM image of tungsten net-pattern grid die with 3 mm in diameter. (**b**) SEM secondary electron image of Hf-based amorphous alloy showing an macro view of the imprinted rectangular shape patterns produced by depression of amorphous alloy with compressive loading; inset image shows macro overall view of patterned Hf-based amorphous alloy. (**c**) Detail view of the SEM image of rectangular patterns imprinted on amorphous alloy showing clear sharp edge of patterns without shear localization. (**d**) Detail view of the SEM image of lateral edge of imprinted on amorphous alloy showing clear sharp depth edge of pattern produced by compressive loading; inset image is three dimensional topological profilemeter image of lateral edge of imprinted on amorphous alloy. (**e**) Profilemeter scanned image of Hf-based amorphous alloy showing an overall view of the imprinted rectangular shape patterns with difference of heights.

A macro view of the structure of the tungsten patterning die, fabricated by an electro-deposition process, with 3 mm in diameter and the thickness with 25 μm used in current study is shown in inset image of Fig. 4(a), while Fig. 4(a) shows a zoomed in view of the tungsten net-pattern grid die showing the edge of rectangular pattern in which the square opening is 97 × 97 μm^2^ in size and the struts are 25 μm wide. Figure 4(b) presents a macro-view of the imprinted grid pattern on the amorphous alloy surface fabricated in 7 mm^2^ areas by cold-plastic forming via compressive loading. A detailed view of the patterned area in Fig. 4(c) shows well-aligned rectangular-shaped patterns with clear edges transferring the three-dimensional shape from the tungsten die to the glassy alloy without any evidence of shear bands or cracks. Figure 4(d), which corresponds to a zoomed-in SEM image of the lateral edge of patterned area, shows the surface of the glassy alloy deformed normal to the surface direction with 22 μm depth (marked by arrow) which is similar to the thickness of tungsten die. The amounts of the homogeneous deformation during cold plastic forming can be seen by inset image of Fig. 4(d), which corresponds to a profilemeter scan of a corner of the lateral pattern. The severely deformed amorphous specimen shows a distinct height difference while maintaining the smooth surface of the as-cast state with the sharp edges of the patterns without any cracks or other observable shear bands. To further quantify the deformation of imprinted Hf-based amorphous alloy, we performed surface topological analysis by profilemeter. Figure 4(e) shows a scanned image of the imprinted Hf-based amorphous alloy surface, where an overall view of the imprinted pattern shapes can be clearly seen. The depth profile obtained from the profilemeter results in Fig. 4(e) shows difference of height of the imprinted region and the amount of deformation is the largest in lateral region (*Δh* = 22 μm) compared to the deformation amount of rectangular patterned region (*Δh* = 2 μm) corresponding to net-pattern area of tungsten grid.

We believe that the difference of loading pressure at the contacting surface (applied loading area) between amorphous alloy and die during deformation is the reason for the difference of the imprinted heights. In general, the stress is not uniformly distributed over the area. The external load or pressure (*p*) is balanced by the internal resisting force 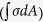, where σ is the stress normal to the surface and A is the cross-sectional area of contact surface^1^. When the volume remains constant during plastic deformation, the strain is defined also to the change in area referred to the initial area 
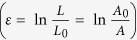
1. Therefore, the large contact area of amorphous to die in lateral part of tungsten grid can plastically deform much more compared to the strut part of tungsten grid which has smaller contact area.

Kumar *et al*. quantitatively evaluated the effect of pressure and interfacial properties on the nanoimprinting of bulk metallic glasses and revealed the pressure results in a highly inhomogeneous distribution at the center and edge of sample^36^. For macroscopic sample sizes and typical applied load, the normalized pressure is expressed by


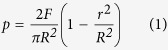


when load grows linearly with time, *F* = *βt* (where *β* is the loading-ramping rate, *t* is total processing time in sec.), *r* is the radial coordinate and *R* is the sample radius.

Understanding the filling dynamics is important for obtaining well-controlled microstructured surfaces via imprinting, the cavity-filling velocity can be described using the Hagen-Poiseuille expression,


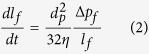


where *l*_*f*_ is the penetration depth, *η* is the viscosity, *d*_*p*_ is the cavity diameter, *ΔP*_*f*_ is the pressure for driving flow into the cavity (*ΔP*_*f*_ = *p* + *p*_*σ*_, where *p*_*σ*_ associated with the interfacial tension *σ* of the BMG front in the cavity), where capillary stresses on the cavity-filling dynamics can be excluded due to the macro-size cavities.

In the absence of capillary stresses, *p*_*σ*_ = 0. The normalized filling ratio, *λ* defined by factoring out the ratio of the final disk radius *R*_*T*_ to the equivalent sphere radius *R*_*0*_, expressed


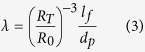


Evolution of the pressure distribution in a BMG disk processed at linear-load-ramping condition is expressed by


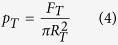


where *T* is final time, *P*_*T*_ is normalized average pressure at time *T*, *F*_*T*_ is the final load and *R*_*T*_ is the final radius of the disk. These pressure inhomogeneous between edge and center of sample can be explained the observed variation in filling depth shown in Fig. 3(d,e) resulting in difference of depth profile at the final process between lateral region and rectangular region.

**Figure 5:**
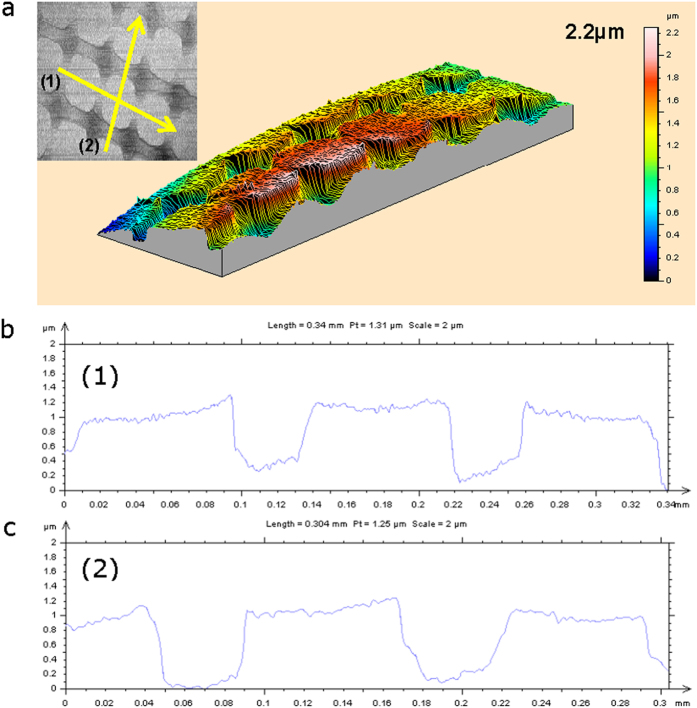
(**a**) Three dimensional profilemeter image of surface topology analysis obtained from the rectangular patterns imprinted on Hf-based amorphous alloy; inset profilemeter image shows analyzed regions scanned following the yellow line from left, bottom to right, top beginning at numbering position. (**b**) Height intensity profile extracted from the linescan analysis corresponding to line (1) in Fig. 5a. (**c**) Height intensity profile extracted from the linescan analysis corresponding to line (2) in Fig. 5a.

Figure 5(a) shows regional scanned 3-dimensional surface topology image obtained from the rectangular patterns imprinted on Hf-based amorphous alloy. The inset profilemeter image shows both regional and line scanning, respectively. The line scans performed following the yellow line from left (number 1), bottom (number 2) to right, top beginning at numbering position. Figure 5(b,c) show height intensity profiles extracted from the line scan analyses corresponding to line (1) and line (2), respectively. The maximum depth difference obtained from surface topological scanning analyses is 2.2 μm. However, as shown in Fig. 5(b,c) line scanned results, the depth difference at the center of the rectangular imprinted pattern is ≤1.2 μm.

**Figure 6:**
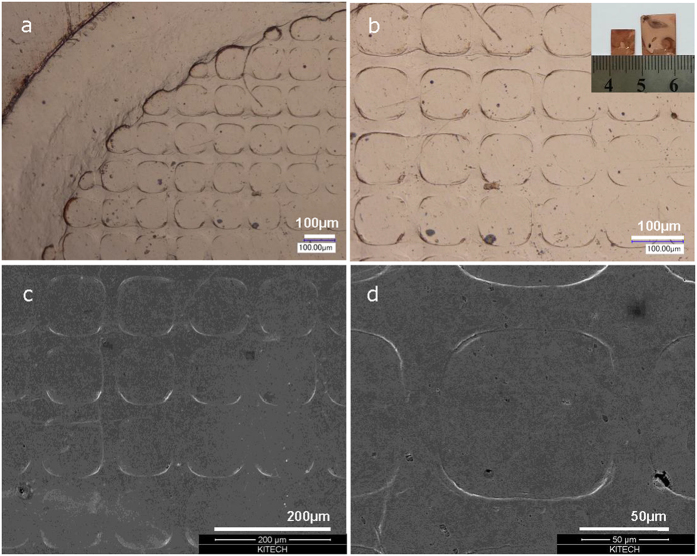
(**a**) Optical image of patterned Cu foil showing an macro view of the imprinted rectangular shape patterns produced by depression of Cu foil with compressive loading of patterned Hf-based amorphous alloy as die. (**b**) Detail view of the optical image of imprinted on Cu foil showing clear edge of pattern produced by compressive loading. (**c**) SEM image of rectangular patterns imprinted on Cu foil showing macro view of the imprinted rectangular shape patterns produced by depression of Cu foil. (**d**) Detail view of the SEM image of lateral edge of imprinted on Cu foil showing clear sharp edge of pattern by compressive loading.

**Figure 7:**
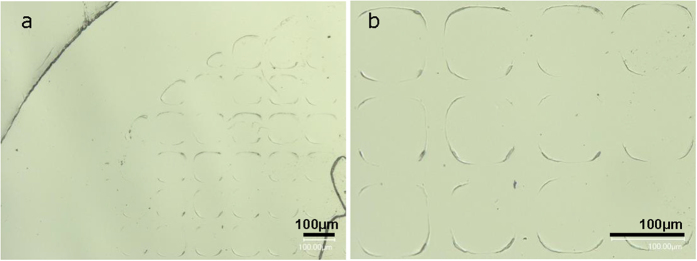
(**a**) Optical image of patterned Zr-based metallic glass showing a macro view of the imprinted rectangular shape patterns produced by depression of Zr-based metallic glass with compressive loading of patterned Hf-based amorphous alloy as die. (**b**) Detail view of the optical image of imprinted on Zr-based metallic glass showing clear edge of pattern produced by compressive loading.

Beyond being imprinted itself, the patterned Hf-based bulk amorphous alloy can be used as a die to imprint patterning features on another material at room temperature. The optical images of the imprinted Cu foil used in the present example are shown in Fig. 6(a,b). The SEM images in Fig. 6(c,d) show detail view of rectangular patterns imprinted on Cu foil showing clear sharp edge of patterns produced by depression of Cu foil, which displays inverted replica of the patterned bulk amorphous alloy features, shown in Fig. 4(b). Moreover, even other amorphous alloy can be imprinted, Fig. 7. The optical images in Fig. 7(a,b) show the rectangular patterns imprinted on Zr-based metallic glass via the Hf-based glass die. Clear sharp edges of patterns produced by the depression of Zr-based metallic glass show the inverted replica of the patterned Hf-based bulk amorphous alloy just as the Cu foil, shown in Fig. 6. The results summarized in Fig. 3 demonstrate the ability of amorphous alloys or metallic glasses to precisely replicate patterning features onto both the conventional metals and the other amorphous alloys.

The deformation map for BMG developed by Spaepen describes two modes of deformation, homogeneous and inhomogeneous flow^4^. The boundary line between the homogeneous and inhomogeneous flow regions on the map is calculated by the general flow equation. In the case of homogeneous deformation, this leads to Newtonian viscous behavior, and an expression for the viscosity can be derived by


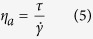


where the equivalent shear stress (*τ*) and shear strain rate 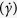 have been calculated from the uniaxial values by the von Mises criterion, (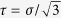: 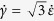) where *σ* is the normal stress and 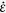 is the strain rate. The apparent viscosity of the MG at RT can be estimated by


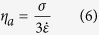


**Figure 8:**
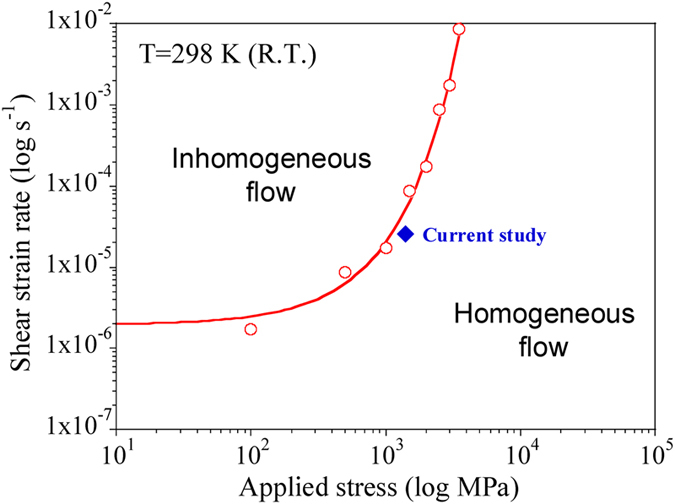
Stress-strain rate dependence of homogeneous and inhomogeneous flow, calculated from the theoretical models in ref. 4.

At 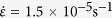, the steady state flow stress *σ* is 1453 MPa, and the apparent viscosity *η*_*a*_ of the MG is about 3.2 × 10^13^ Pa s. MGs are often considered viscoplastic “liquid metals”, due to their deformation behavior at RT^24^. Based on this these equations, it is now possible to construct a deformation map. Figure 8 shows the stress-shear strain rate dependence of homogeneous and inhomogeneous flow at RT, based on the Spaepen’s models. The boundary line on the deformation map can be calculated from the stress exponent (ratio between shear strain rate and stress). In this map the stress and shear strain rate axes are extended further than normal conditions to show that if we apply a stress of ~2000 MPa at a strain rate below 9 × 10^−5^ s^−1^ to typical BMGs, they theoretically can be homogeneously deformed. The FEM (Finite Element Method) simulations (shown in Supplement 1) were performed to analyze the quantitative difference between the deformation behavior of inhomogeneous and homogeneous regimes in BMG. As shown in the simulation results, the homogeneous deformation is mainly dependent of shear strain rate rather than effective strain. Moreover, it is expected that the transition from localized or inhomogeneous deformation to homogeneous flow at RT should also depend on the elastic properties (i.e., shear modulus, viscosity, etc.) of MGs.

## Discussions

Johnson *et al*. have reported that the viscosity-shear modulus relationship implies that softening of metallic glass-forming liquids induced by either thermal excitation or mechanical deformation is governed by the dependence of shear modulus on the potential energy^21^. Moreover, it is believed that deformation of amorphous materials occurs at specific sites where atomic clusters are able to rearrange themselves in response to applied stresses^5^,^32^. In the potential energy landscape theory, a potential energy function is used to model the energetic landscape of the system, which comprises a population of inherent states (basins) of the liquid associated with local minima^21^,^30^,^37^,^38^. The strain rates are expressed in units proportional to the frequency of oscillation about the minimum in the interatomic or intermolecular potential^30^. It has been shown that the appearance of a multidimensional potential energy landscape can be related to the form of the interatomic or intermolecular potential. The potential energy difference between minimum and the transition states which are separated by potential energy barrier (height) and the path length (distance)^39^.

The underlying relaxation mechanisms of liquids and glasses are believed to be governed by two kinetic processes: a fast process, termed the β-process, viewed as a locally initiated and reversible process, and a slow process, termed by α-process, viewed as a large scale irreversible rearrangement of material^37^,^40^. In the current study, we assume the relaxation mechanism of amorphous materials is governed by a slow process is α-process rather than β-process, viewed as a large-scale irreversible rearrangement of the material by low strain rate^37^,^40^. For considering the potential energy barrier, the total energy barrier for configurational hopping between inherent states, which can be regarded as the activation barrier for shear flow, can be expresses as


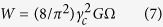


Here, *γ*_*c*_ is the shear strain limit of the material, *Ω* is effective shear transformation zone volume, *G* is effective shear modulus^13^. In case of non-Newtonian flow, the rate of barrier softening can be formulated as


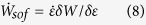


Where 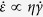 is the rate of dissipated mechanical energy density (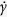 is the strain rate, *η* is a viscosity), which at steady-state can be equivalent to the production rate of specific configurational potential energy density.


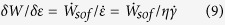


Equation (9) is a thermodynamic parameter denoting changes in *W* with respect to changes in 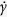. The probability of shear instability by hopping of the energy barrier of atoms or clusters decreases due to increase height of energy barrier and therefore appeases a whole volumetric change of material rather than local shear instability. Moreover, in the case of a two-state system, such as plane strain state, the transition between one state and the other state constitutes an elementary increment of shear strain^30^.

For considering the path length of the interatomic potential, decreases in distance of path length indicates the disappearance of the local energy minimum, which leads to a mechanical instability or shear localization. The regions of mechanical instabilities could be associated with the distribution of the atomic displacements (or rearrangement)^30^,^31^. The region of rearrangement taking place without significantly affecting the relative positions of molecules in the environment of the transforming region is defined as STZ^30^,^38^. The instability path length can be considered the size of STZ, the estimation of degree of localization of the mechanical instability described by Lacks *et al*. who proposed that the participation number is not proportional to system size, suggesting the instabilities are localized. However, for the delocalized motion the participation number is proportional to the system size^31^. Therefore, bulk amorphous material with large number of atomic clusters is inclined to deform via delocalization rather than localization due to the suppression of instability under low strain rate. As a result, homogeneous deformation will be the controlling deformation mechanism instead of shear localization. It was reported that dependence of the shear modulus and yield stress on variations of temperature or shear strain rate are equivalent, therefore, the equilibrium distribution of STZ’s by temperature is the same as the strain-induced equilibrium distribution of STZ^21^,^32^.

The effect of shear strain rates on the effective shear modulus is possible to distinguish as the system has sufficient time to relax thermally towards the new local minimum-energy configuration at low strain rate. For slow strain rates, the time scales for thermally activated structural relaxation (α-process) are smaller than the reciprocal shear rate 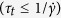; the material flows similar to temperature above T_g_ corresponding to a liquid-like regime^32^. An increase in temperature with constant strain rate leads to the same macroscopic behavior of the system as smaller strain rates and constant temperature. Time and temperature are equivalent parameters and lead to the same mechanical response of amorphous system, as long as local mechanical stability is maintained^32^. It is theoretically proposed that a wide range of viscoplastic deformation occurring in a simple molecular-dynamics model of amorphous solids at low temperature^30^. It is also represented that the possibility of free volume generation in amorphous matrix with no shear bands at RT^22^.

## Conclusions

We present that patterning of a typical brittle Hf-based amorphous alloy and transferring patterns on Cu foil and Zr-based metallic glass by imprinting technique. As demonstrated by experiments in current study, it is possible by cold-plastic forming which can show good micro-viscous deformability at RT without thermal energy. Our work is suggestive of a solution to the problem of embrittlement of glassy alloys during thermoplastic forming and implies it is possible to control the deformation behavior of bulk amorphous alloys at RT, which has broad implications for their RT ductility and even superplasticity. Plastic forming of high strength amorphous alloys at RT resulting from the homogeneous flow of typical brittle amorphous alloy is very important when considering engineering applications.

## Methods

The bulk amorphous alloy chosen for this study is Hf_44.5_Cu_27_Ni_13.5_Ti_5_Al_10_ (at%). Its glass transition temperature T_g_ is 787 K, and thus room temperature deformation (298 K) corresponds to a reduced temperature (T/T_g_) of 0.37, well within the regime for which plastic deformation is expected to be inhomogeneous. Ingots were prepared by arc melting in Ar atmosphere and suction cast into a Cu mold to form 3 mm diameter rods. The amorphous nature of the samples were examined using a Philips APD 3520 X-ray diffractometer with monochromatic Co-Kα radiation (λ = 1.78 Å) and the thermal properties of the samples were measured with a Perkin-Elmer Pyris-1 differential scanning calorimeter (DSC) using continuous heating at a rate of 40 K/min. For the uniaxial compression testing, rod-shaped specimens 3 mm in diameter and 6 mm in height (2:1 height-to-diameter ratio) were prepared by micro-polishing. For cyclic compression testing, specimens were compressed up to 93% of the yield strength of the amorphous alloy (1813 MPa). Uniaxial compression tests were performed under quasistatic loading at an initial strain rate of 3 × 10^−4^ s^−1^ at room temperature. For the patterning experiments, a rectangular, 4 mm in width and 80 mm long, bar was cast in a water-cooled copper mold. 10 samples 2 mm in height cut (h/a ratio = 0.5) were cut by diamond saw and the ends of the samples were carefully polished flat and normal to the longitudinal axis (patterning axis). Patterning was performed under the same compressive loading condition as the uniaxial compression using a tungsten grid (Pacific Grid-Tech W-200) which has 3 mm diameter, 97 μm in grid spacing and 25 μm in thickness. For imprinting of pattern from the Hf-based glass onto other materials, a rectangular, 10 mm in width and 30 μm, 100 μm in thickness Cu foils and Zr_55_Cu_20_Ni_10_Al_10_Ti_5_ (at%) metallic glass with 100 μm in thickness were prepared, respectively. The samples were uniaxially loaded between the WC platens (6 mm in thickness with 38 mm in diameter) at a strain rate of 1.5 × 10^−5^ s^−1^ (Instron type electro mechanical testing system) up to 75% of the yield strength of amorphous alloy (1453 MPa) at which they were held for period of 21 hours. The imprinting conditions calculated by distribution of effective strain with the shear strain rate, the shear stress does not exceed the yield stress of MG at given stress level. Surface topological analysis was performed with a Hommelwerke T8000 profilemeter.

## Additional Information

**How to cite this article**: Kim, S.-Y. *et al*. Imprinting bulk amorphous alloy at room temperature. *Sci. Rep*. **5**, 16540; doi: 10.1038/srep16540 (2015).

## Supplementary Material

Supplementary Information
