# Supplementary material for: Imprinting bulk amorphous alloy at room temperature
Source: Sci Rep. 2015 Nov 13;5:16540. doi: 10.1038/srep16540 (PMC4643295; doi:10.1038/srep16540)

Imprinting bulk amorphous alloy at room temperature

Song-Yi Kim[1], Eun-Soo Park[2], Ryan T. Ott[3], Thomas A. Lograsso[3], Moo-Young Huh[4], Do-Hyang Kim[5], Jürgen Eckert[6,7] & Min-Ha Lee[1*]

[1] Rare Metals R&D Group, Korea Institute of Industrial Technology, Incheon 406-840, Korea
[2] Global Technology Center, Samsung Electronics Co., Ltd., Suwon, 443-742, Korea
[3] Division of Materials Sciences and Engineering, Ames Laboratory (USDOE), Ames, Iowa 50011, USA
[4] Department of Materials Science and Engineering, Korea University, Seoul, 136-701, Korea
[5] Department of Metallurgical Engineering, Yonsei University, Seoul, 120-749, Korea
[6] IFW Dresden, Institute for Complex Materials, P.O. Box 27 01 16, D-01171 Dresden, Germany
[7] TU Dresden, Institute of Materials Science, D-01062 Dresden, Germany

Supplement 1

FEM simulation analysis on the effective strain effect by strain rate changing on the formation of shear band in metallic glass during the patterning process. Shear strain rate plays a key role to decide shear banding or homogeneous flow during deformation rather than effective strain.

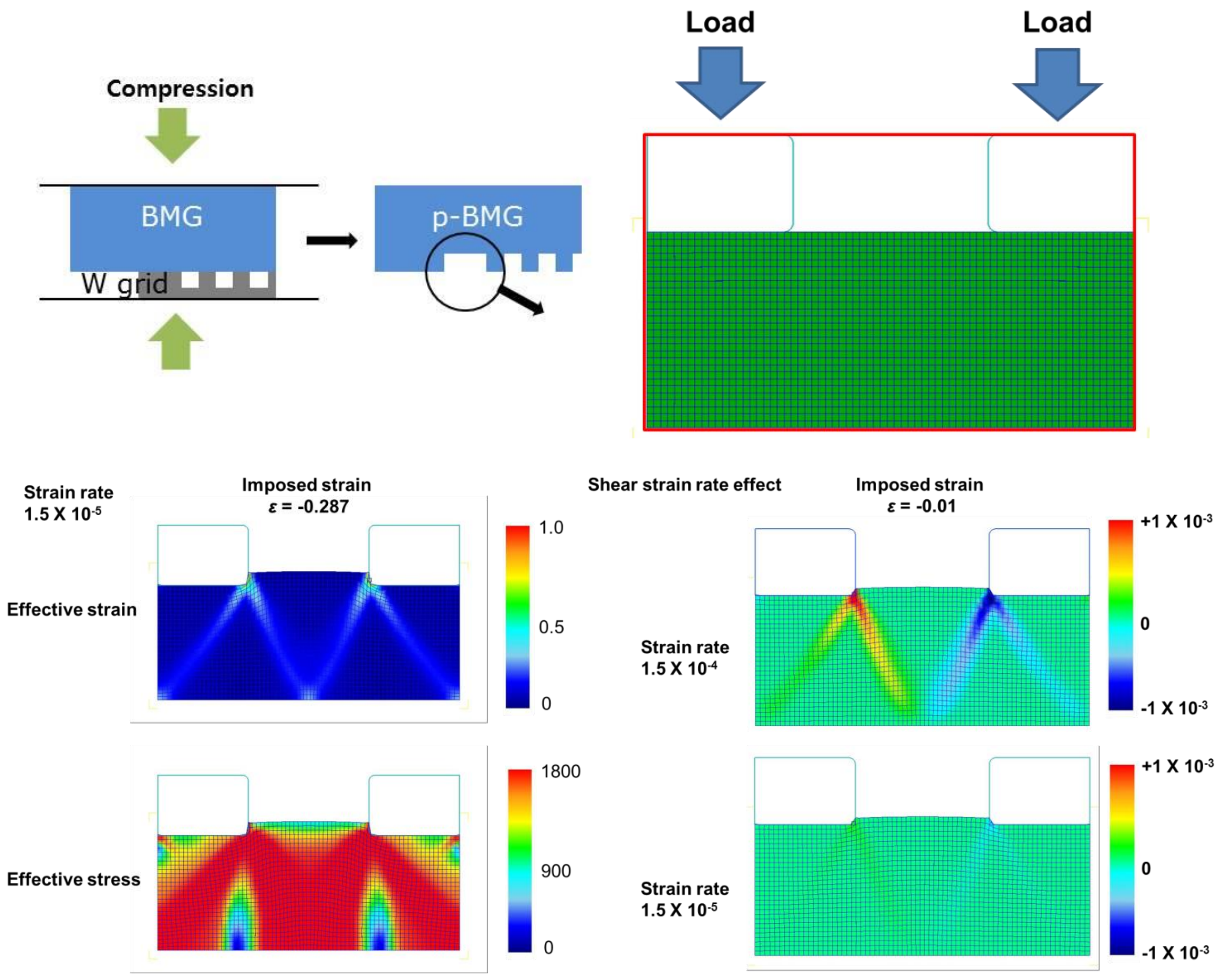

Supplement: Supplementary Information [file srep16540-s1.pdf]
